# Tongue necrosis as a manifestation of immune dysfunction: A complex case of systemic lupus erythematosus, histoplasmosis, and macrophage activation syndrome

**DOI:** 10.1002/ccr3.7735

**Published:** 2023-07-21

**Authors:** Madalyn Walsh, Alick Feng, Petar Lenert, Bharat Kumar

**Affiliations:** ^1^ Department of Internal Medicine University of Iowa Hospitals & Clinics Iowa City Iowa USA; ^2^ Division of Immunology University of Iowa Hospitals & Clinics Iowa City Iowa USA

**Keywords:** histoplasmosis, macrophage activation syndrome, systemic lupus erythematosus, tongue necrosis

## Abstract

Immune dysfunction can manifest in unexpected ways. We present the case of a patient with systemic lupus erythematosus (SLE) in whom the first sign of disseminated histoplasmosis and consequent macrophage activation syndrome (MAS) was tongue necrosis. In those with immune dysfunction, a high index of clinical suspicion for atypical infections is warranted.

## Graphical abstract

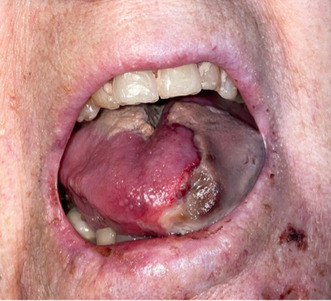

## 1 BACKGROUND

Tongue necrosis is a rare entity as the tongue is a highly vascularized structure supplied by the lingual artery, a branch of the external carotid artery. Tongue necrosis typically presents with unilateral lingual pain, swelling, discoloration and, in severe cases, necrosis. There are many underlying factors that can contribute to the development of tongue necrosis (Table 1).^1^, ^2^, ^3^, ^4^, ^5^, ^6^, ^7^, ^8^, ^9^, ^10^, ^11^, ^12^, ^13^, ^14^, ^15^, ^16^, ^17^, ^18^, ^19^ Giant cell arteritis (GCA), ANCA (Anti‐Neutrophil Cytoplasmic Antibody)‐associated vasculitis and other vasculitides have been previously described as underlying systemic causes of tongue necrosis.^1^, ^2^, ^3^, ^4^ Other potential causes include infection (particularly tuberculosis and syphilis), malignancy, radiation therapy, use of vasoconstricting medications and systemic hypoperfusion such as in the setting of shock.^1^ This case report adds histoplasmosis to this list of differential diagnoses.

**TABLE 1:** A summarization of the differential diagnosis for tongue necrosis, specific examples of causative factors, and signs and symptoms associated with each entity.

Differential diagnosis in tongue necrosis	Example of specific disease or agent	Associated clinical signs and symptoms
Systemic vasculitides	Giant cell arteritis,^1^, ^2^, ^3^, ^4^ ANCA vasculitis^5^	Tongue pain, macroglossia, headache, rash, vision changes, fever, hematuria
Infection	Fungus,^6^, ^7^, ^8^ syphilis,^9^ tuberculosis^10^	Fever, leukocytosis, cough, shortness of breath
Malignancy	Lymphoma,^11^ sarcoma,^12^ carcinoma^13^	Lymphadenopathy, soft tissue mass, fatigue
Radiation therapy	Radiation arteritis^14^	Localized tissue damage
Cardiovascular event	Myocardial infarction,^15^ embolism,^16^ hypoperfusion^17^	Chest pain, hypovolemic shock
Drugs	Chemotherapy,^18^ vasopressin^19^	Digital ischemia, Raynaud phenomenon

## 2 CASE REPORT

**FIGURE 1:**
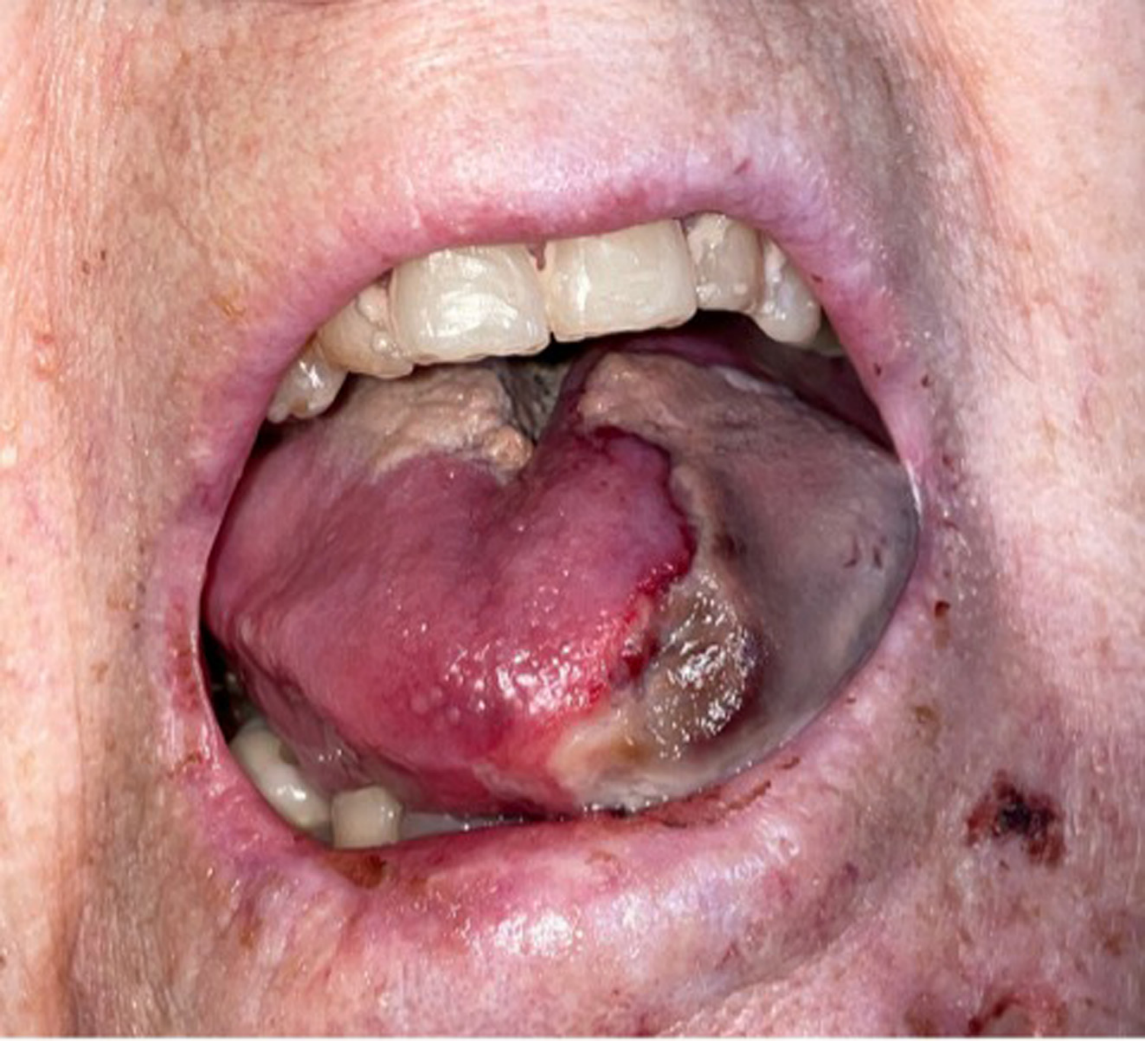
Photograph of left sided tongue necrosis present on hospital admission.

A 65‐year‐old female presented with left sided tongue necrosis (Figure 1). She had a past medical history significant for SLE on mycophenolate mofetil (MMF) 1000 mg twice daily and prednisone 5 mg daily, Factor V Leiden, and atrial fibrillation. The patient initially developed pain and numbness on the left side of her tongue which worsened over the course of several weeks and prompted her to see her primary care doctor. She was started on amoxicillin‐clavulanate without improvement of her symptoms. A Computed Tomography (CT) scan was performed and revealed acute inflammatory phlegmonous changes of the left tongue without any abscess or fluid collection as well as left anterior lateral facial soft tissue and left submandibular space inflammation.

On admission, vital signs included blood pressure 106/68 mmHg, pulse 72 beats per minute, temperature 35.8°C, oxygen saturation 95%. Physical exam of the oral cavity revealed a left sided area of tongue necrosis that extended medially and did not cross midline (Figure 1). There was also a fissure in the mouth posteriorly as well as erythema of the lateral lower lip. No thrush was noted. While initially afebrile upon admission, the patient became febrile on day 3 of hospitalization and continued to be intermittently febrile until her demise.

Vasculitides including GCA, polyarteritis nodosa, ANCA‐associated vasculitis, and cryoglobulinemic vasculitis were considered as possible etiologies. PET‐CT scan was obtained and did not show any evident signs of vessel inflammation. ANCA titers and cryoglobulin levels were also within normal limits. Laboratory examination revealed a soluble interleukin 2 receptor (sIL2r) 11,141 U/mL (N 137–838 U/mL), ferritin 42,975 ng/mL (N 13–150 ng/mL), C‐Reactive Protein (CRP) 19.5 mg/dL (N < 0.5), fibrinogen <50 mg/dL (N 194–448 mg/dL). Additional lab values on admission were significant for C3 Complement level was 47 mg/dL (N 90–180 mg/dL), C4 complement was 4 mg/dL (16–47 mg/dL), white blood cell count of 2.8 K/MM3 (N 3.7–10.5 K/MM3), hemoglobin of 8.5 g/dL (N 11.9–15.5 g/dL), platelet count of 47 K/MM3 (N 150–400 K/MM3). Erythrocyte sedimentation rate (ESR) was 15 mm/Hr (N 0–20 mm/Hr). Antinuclear antibody (ANA) was <1:80 (N < 1:80). Double‐stranded DNA antibody (dsDNA) was negative. Given negative dsDNA and lack of clinical evidence of SLE exacerbation, it was felt to be unlikely that this patient's presentation was secondary to SLE alone.

Infectious work up was performed with bronchoalveolar lavage (BAL) confirming disseminated histoplasmosis. Histoplasma urine and blood antigens were positive at >25.0 ng/mL (N undetectable) and > 20.0 ng/mL (N undetectable) respectively. Blood cultures were obtained and revealed fungemia with speciation showing histoplasma. Tongue and lip biopsies revealed necrotic tissue with neutrophilic inflammation and yeast forms consistent with histoplasma based on Periodic acid–Schiff (PAS) and Grocott methenamine silver (GMS) stains. Bone marrow biopsy was obtained and revealed hemophagocytosis and histoplasmosis.

Malignancy was also considered given the location of necrosis as well as severe leukocytopenia and thrombocytopenia but neither flow cytometry of both the blood and bone marrow nor superficial tongue biopsies revealed evidence of malignancy. The patient had a past medical history significant for Factor V Leiden, a mutation in part of the coagulation cascade which results in an increased risk for venous thromboembolism, as well as atrial fibrillation which increases risk of embolization events. COVID‐19 nucleocapsid and spike‐protein antibodies were also obtained with the anti‐spike antibody positive, indicating that the patient had previously been vaccinated, and the anti‐nucleocapsid antibody negative, indicating that she had not had a recent infection that could have resulted in a hypercoagulable state.

The patient was started on liposomal amphotericin 3 mg/kg for antibiotic coverage but continued to have fevers and rising ferritin levels, raising concern for cytokine storm given her underlying autoimmune disease and active systemic fungal infection. The patient was also noted to have low fibrinogen <50 mg/dL (N 194–448 mg/dL). And dramatically rising ferritin 42,975 ng/mL (N 13–150 ng/mL) in addition to her cytopenias. Serum soluble interleukin 2 receptor (sIL2r) was also markedly elevated to 11,141 U/mL (N 137–838 U/mL). Triglycerides were elevated to 377 mg/dL (N < 150 mg/dL). Flow cytometry of peripheral blood showed increased CD38^++^/HLA‐DR/CD8^+^ which was highly suggestive of hemophagocytic lymphohistiocytosis (HLH). Bone marrow biopsy was performed 1 week after initial presentation and confirmed both hemophagocytosis and histoplasmosis. The diagnostic criteria for HLH requires 5 out of 9 findings be present, of which our patient had 6. These criteria and the corresponding findings in our presenting case are listed in Table 2.^20^ Laboratory markers and clinical picture initially did improve with the initiation of steroids, IVIG 2 g/kg total over 4 days and Anakinra 100 mg daily along with concurrent treatment of histoplasmosis. However, following stabilization for 2–3 days, she developed septic shock and passed away.

**TABLE 2:** Diagnostic criteria for HLH and the criteria met by the patient in the case being presented.

Diagnostic criteria for HLH^20^	Presenting case
Fever ≥38.5°C	Intermittently febrile with maximum temperature of 38.9°C
Splenomegaly	Not present
Cytopenias, need 2/3: hemoglobin <9 g/dL; platelets <100,000/μL; absolute neutrophil count <1000/microL	Hemoglobin of 8.5 g/dL Platelets 47 K/MM3
Hypertriglyceridemia (fasting triglycerides >265 mg/dL) or hypofibrinogenemia (fibrinogen <150 mg/dL)	Triglycerides 377 mg/dL (N < 150 mg/dL) Fibrinogen <50 mg/dL (N 194–448 mg/dL)
Hemophagocytosis in bone marrow, spleen, lymph node, or liver	Present on bone marrow biopsy
Low or absent NK cell activity	Not tested
Ferritin >500 ng/mL	Peak ferritin 42,975 ng/mL (N 13–150 ng/mL)
Elevated soluble CD25 (soluble IL‐2 receptor alpha [sIL‐2R])	sIL2r 11,141 U/mL (N 137–838 U/mL)
Elevated CXCL9	Not tested

## 3 DISCUSSION

To our knowledge, this is the first documented case in peer‐reviewed literature of tongue necrosis secondary to histoplasmosis. Histoplasmosis is the most common endemic mycosis found in the United States and is the most likely fungus to cause infection resulting in hospitalization.^21^ Most individuals exposed to histoplasma spores develop a subclinical infection and are asymptomatic. Those that develop acute symptoms typically present with pulmonary manifestations such as dyspnea and cough.^22^ Disseminated disease may occur in patients who have a particularly high level of exposure or have immune dysfunction, such as those with autoimmune disease, those taking immune modulators, and those with inborn errors of immunity.^23^

While cases of tongue necrosis have been well described in the setting of GCA, there is a paucity of literature on the occurrence of tongue necrosis as a result of fungal infection, and none published that are specifically the result of histoplasma infection.^1^, ^2^, ^3^, ^4^ Tongue necrosis has been seen in the setting of mucormycosis in three published cases (Table 3). These three patients had conditions that resulted in immune dysfunction, predisposing them to fungal infection.^6^, ^7^, ^8^ Our patient would also be considered as having immune dysfunction in the setting of SLE with history of high disease activity and long‐term steroid and MMF administration. Specifically, both SLE and the MMF used to treat SLE are associated with dysfunction in T‐cell activity with the mechanism of action of MMF being the reversible inhibition of inosine monophosphate (IMP) dehydrogenase, leading to decreased B and T‐cell proliferation as well as decreased antibody production. Because the Th17 subset of T‐cells is necessary for coordinating and regulating neutrophilic clearance of fungal infections, this is likely a root cause for this patient's susceptibility to histoplasmosis and severity of infection. The additional challenge is that T‐cells play important roles in resolving inflammatory states. After all, the three previously reported cases of mucormycosis‐induced tongue necrosis were associated with conditions that resulted in impaired neutrophil‐mediated inflammation. In this case, we speculate that T‐cell dysfunction led to a paradoxical state in which there were elements of both immune deficiency (systemic fungal infection) and hyperinflammation (HLH/MAS). This, in turn, raises a clinical dilemma in immunomodulatory treatment. We elected to administer IVIG and anakinra since they are unlikely to impair T‐cell defenses, and there may be a protective element of IVIG when administering high‐dose steroids to treat the cytokine storm. Nevertheless, the patient's burden of disease led to progression of sepsis to shock.

**TABLE 3:** Cases of tongue necrosis caused by fungal infection.

Case number	Year	Age, years	Sex	Fungus	Risk factors	Antibiotic therapy	Outcome
1^6^	2011	69	Female	Mucormycosis	Type 1 Diabetes mellitus	Granulocyte infusions, gentamicin, clarithromycin, voriconazole, amphotericin B	Survived
2^21^	2014	82	Female	Mucormycosis	Aplastic anemia, antilymphocyte globulin and cyclosporin	Amphotericin B, posaconazole	Died
3^22^	2018	56	Female	Mucormycosis	Type 1 Diabetes mellitus, diabetic ketoacidosis, active dengue fever infection	Ceftriaxone, amphotericin B	Survived
Presenting case	2022	65	Female	Histoplasmosis	Metronidazole, ceftriaxone	Metronidazole, ceftriaxone	Died

Tongue necrosis and cytokine storm (HLH/MAS) share common predisposing triggers, including infection, malignancy, and autoimmune disease. We believe histoplasmosis was the trigger for cytokine storm in our patient in the setting of T‐cell dysfunction secondary to SLE and immune modulation. Laboratory findings, radiographic studies, and histopathology allowed us to exclude malignancy, thromboembolism, and vasculitis from our list of differential diagnoses for the cause of tongue necrosis. Treatment of the infection and the hyperinflammation resulted in immediate improvement but the prognosis of disseminated histoplasmosis remained poor. This case highlights the importance of maintaining a high index of clinical suspicion for fungal infection in those with immune dysfunction.

## AUTHOR CONTRIBUTIONS

**Madalyn Walsh:** Conceptualization; data curation; formal analysis; investigation; project administration; resources; writing – original draft; writing – review and editing. **Alick Feng:** Resources; supervision; validation; writing – review and editing. **Petar Lenert:** Investigation; resources; supervision; writing – review and editing. **Bharat Kumar:** Formal analysis; resources; supervision; writing – review and editing.

## FUNDING INFORMATION

Funding was received from the University of Iowa.

## CONFLICT OF INTEREST STATEMENT

The authors have no conflicts of interest to disclose.

## ETHICS STATEMENT

Ethical approval is not required by the Institutional Review Board for case report publication.

## CONSENT

Written informed consent was obtained from the patient to publish this report in accordance with the journal's patient consent policy.

## Data Availability

Data sharing is not applicable to this article as no new data were created or analyzed in this study.
